# 气管内超声引导下经支气管针吸活检在肺外肿瘤胸内转移中的诊断价值

**DOI:** 10.3779/j.issn.1009-3419.2015.05.07

**Published:** 2015-05-20

**Authors:** 加源 孙, 亮 鲍, 家俊 滕, 润波 钟, 薇琼 翁, 琴 张, 宝惠 韩

**Affiliations:** 1 200030 上海，上海交通大学附属胸科医院内镜室 Department of Endoscopy, Shanghai Chest Hospital, Shanghai Jiaotong University, Shanghai 200030, China; 2 214002 无锡，江苏省无锡市第二人民医院呼吸科 Department of Respiratory Medicine, Wuxi Second People's Hospital, Wuxi 214002, China

**Keywords:** 肺外肿瘤, 支气管超声, 针吸活检, 免疫组织化学, Extrapulmonary neoplasm, Endobronchial ultrasound, Needle aspiration, Immunohistochemistry

## Abstract

**背景与目的:**

气管内超声引导下经支气管针吸活检（endobronchial ultrasound-guided transbronchial needle aspiration, EBUS-TBNA）已广泛应用于增大纵隔及肺门淋巴结的诊断，本研究旨在进一步评价EBUS-TBNA对肺外恶性肿瘤胸内转移的诊断价值和安全性。

**方法:**

前瞻性入组既往诊断/新发肺外恶性肿瘤、影像学检查提示肺内病变和/或胸内淋巴结增大怀疑为肺外恶性肿瘤胸内转移所致，需行EBUS-TBNA取得病理诊断的患者。

**结果:**

共41例患者入组，穿刺纵隔/肺门淋巴结67组，肺内肿块5例。EBUS-TBNA明确诊断肺外肿瘤胸内转移14例，原发性肺癌10例，反应性淋巴结炎9例，类结节病反应4例，结核1例；假阴性3例。EBUS-TBNA对肺外肿瘤胸内转移诊断的敏感性和准确性分别为87.50%和95.12%。18例来源或类型不明的恶性肿瘤中，结合免疫组化检测，明确诊断肺外肿瘤胸内转移12例和原发性肺癌6例。

**结论:**

EBUS-TBNA是诊断肺外肿瘤胸内转移一种安全、有效的方法，免疫组化检测结果可为鉴别肺外肿瘤胸内转移和原发性肺癌提供有效依据。

## Abstract (en)

**Background and objective:**

Endobronchial ultrasound guided transbronchial needle aspiration (EBUS-TBNA) has been widely applied in diagnosing mediastinal and hilar adenopathy. This study is further to evaluate value and safety of EBUS-TBNA in diagnosing intrathoracic metastasis from extrapulmonary malignancy.

**Methods:**

Prospectively analysis of 41 patients suspected intrathoracic metastasis from previous diagnosed/concurrent extrapulmonary malignancies in Shanghai Chest Hospital, with radiologic findings showing mediastinal/hilar lymph node enlargement or intrapulmonary lesion requiring EBUS-TBNA examination for pathological diagnosis.

**Results:**

41 candidate patients enrolled, and 67 mediastinal/hilar lymph nodes and 5 intrapulmonary lesions were aspirated. 14 intrathoracic metastasis, 10 primary lung cancer, 9 reactive lymphadenitis, 4 sarcoid-like reactions, and 1 tuberculosis was diagnosed by EBUS-TBNA. Sensitivity and accuracy of EBUS-TBNA in diagnosing intrathoracic metastasis was 87.50% and 95.12%, respectively. Immunohistochemistry (IHC) was performed in 18 malignant tumors to obtain definite type or origin, twelve intrathoracic metastasis and 6 primary lung cancer were further confirmed.

**Conclusion:**

EBUS-TBNA is a safe, effective method for the diagnosis of intrathoracic metastasis from extrapulmonary malignancy. IHC can provide additional evidence for distinguishing extrapulmonary malignancy from primary lung cancer.

恶性肿瘤可以通过血道和淋巴结系统进行转移播散，而肺和纵隔是较常见的转移部位^[1]^。在临床工作中，对于既往诊断/新发的肺外恶性肿瘤患者出现孤立的肺内结节、纵隔/肺门淋巴结肿大时，需要积极取得病理学依据以明确病变性质进而指导制定后续的最佳诊疗方案^[2, 3]^。气管内超声引导下经支气管针吸活检（endobronchial ultrasound-guided transbronchial needle aspiration, EBUS-TBNA）自2002年开始研发，目前在肺癌诊断和分期、肺结节病、肺结核等方面的应用价值已得到广泛证实^[4-8]^，但其在肺外肿瘤胸内转移中的诊断价值尚未明确。本研究前瞻性入组上海市胸科医院怀疑肺外肿瘤胸内转移的患者，探讨EBUS-TBNA在肺外肿瘤胸内转移诊断中的价值和安全性。

## 1 对象与方法

### 1.1 病例入选标准

① 既往诊断/新发的肺外恶性肿瘤患者；②胸部电子计算机断层扫描（computed tomography, CT）提示纵隔/肺门淋巴结肿大（短径≥1 cm）和/或位于气管或支气管周围的肺内肿块，或正电子发射计算机断层显像（positron emission tomography/computed tomography, PET/CT）提示纵隔/肺门淋巴结和/或气管或支气管周围的肺内肿块氟代脱氧葡萄糖（fluoro deoxyglucose, FDG）摄取值（standardized uptake value, SUV）增高（SUV≥2.5）；③临床怀疑胸内病变为肺外恶性肿瘤转移所致，需行EBUS-TBNA取得病理诊断者；④无EBUS-TBNA检查的禁忌证。所有患者术前签署知情同意书，本研究经上海市胸科医院伦理委员会批准。

### 1.2 EBUS-TBNA操作

EBUS-TBNA由三位医生操作完成^[9]^。所有患者均在清醒镇静（咪达唑仑）和局部麻醉（利多卡因）下进行^[10]^。先经口行普通支气管镜检查，然后使用搭载电子凸阵扫描的超声支气管镜（BF-UC260F-OL8, Olympus）检查目标病变和周围血管，淋巴结检查根据国际分期标准^[11]^。超声顶端安放水囊，扫描频率7.5 MHz，超声影像的加工通过超声图像处理装置（EU-C2000, Olympus），冻结超声图像的情况下记录目标病变直径，穿刺之前启用多普勒功能排除穿刺针穿入血管，使用22号穿刺吸引针（NA-201SX-4022, Olympus）在实时超声指导下进行穿刺吸引，确认穿刺针进入靶区后，来回移动穿刺针进行抽吸。推荐对目标淋巴结和肿块进行3次穿刺，若已获取足够的组织标本，可穿刺2次。并未采用现场细胞学方法进行细胞学涂片的验证。细胞学涂片检查由两位有经验的细胞病理医生盲目完成，得到的组织条标本经甲醛固定，石蜡包埋制切片后行组织学检查。在对比肺外恶性肿瘤原发部位病理标本进行形态学诊断的基础上，必要时行免疫组化检测，进一步完善诊断。由操作医生判断气管镜检查标本是否行微生物学检查（革兰氏染色、抗酸染色、细菌培养、结核分枝杆菌培养等）。

### 1.3 数据收集及TBNA结果判断

数据被前瞻性记录，包括患者临床资料、普通气管镜表现、EBUS-TBNA穿刺部位、病变直径、穿刺次数、并发症、EBUS-TBNA诊断结果和最终诊断。普通气管镜下表现分为4类：Ⅰ类：无管腔内病变；Ⅱ类：外压性改变不伴粘膜改变；Ⅲ类：粘膜下病变（水肿、充血、粘膜增厚、支气管管腔狭窄、粘膜征象消失和明显的血管结构）而未见明显的粘膜侵犯；Ⅳ类：明显的粘膜肿瘤侵犯^[8]^。TBNA标本中如见到明确的恶性肿瘤细胞，则为阳性结果；如未见恶性肿瘤细胞，则为阴性结果（包括特异性的良性疾病证据和未取得明确诊断的结果）^[12]^。

TBNA结果均经其后CT引导下的经胸针吸活检、淋巴结穿刺或活检、纵隔镜、胸腔镜、开胸手术或至少1年的临床随访验证，患者根据相应的检查结果进行治疗。同一例患者任何一个部位TBNA结果判定为阳性，则认为TBNA总结果阳性；若所有部位TBNA结果均为阴性，则认为TBNA总结果阴性。TBNA阳性诊断经其他病理学结果或临床随访证实为恶性肿瘤，则为真阳性结果，未经证实则为假阳性结果；TBNA阴性结果经其他病理学结果或微生物学结果或临床随访证实为非恶性肿瘤，则为真阴性结果，如被证实为恶性肿瘤则为假阴性结果。

### 1.4 统计学分析

应用SPSS 11.5统计软件对数据进行分析，根据标准定义计算敏感度、特异度、阳性预测值、阴性预测值和准确率。

## 2 结果

### 2.1 患者情况、普通支气管镜表现及EBUS-TBNA穿刺结果

2010年2月-2013年3月共有41例患者入组，其中男性17例，女性24例，年龄22岁-74岁，平均56.4岁。41例既往诊断/新发肺外恶性肿瘤患者包括乳腺癌10例，肠癌7例，甲状腺癌和鼻咽癌各5例，食管癌3例，宫颈癌、前列腺癌和胃癌各2例，阴茎癌、肝癌、肾癌、腹壁基底细胞癌、脑瘤各1例。普通支气管镜下表现：Ⅰ类22例，Ⅱ类1例，Ⅲ类17例，Ⅳ类1例（该例患者外院气管镜活检病理阴性，故行EBUS-TBNA检查）。

41例患者共穿刺72组病变，其中淋巴结67例次，包括2R组3例次，4R组18例次，4L组8例次，7组23例次，8组3例次，11L组6例次，11Rs组3例次，11Ri组3例次。肺内肿块5例次，其中右上肺肿块2例次，右下肺肿块1例次，左下肺肿块2例次。超声图像下病变的平均长径为18.50（5.70-37.20）mm，平均短径为14.80（5.30-24.10）mm。每个部位行1次-7次穿刺，平均2.76次。

### 2.2 病理诊断及随访结果

41例患者中最终确诊为肺外转移癌16例、原发性肺癌11例。16例肺外转移癌中，包括乳腺癌胸内淋巴结转移6例，肠癌胸内淋巴结转移3例，鼻咽癌胸内淋巴结转移1例，食管癌胸内淋巴结转移、胃癌胸内淋巴结转移、肝癌胸内淋巴结转移、肾癌胸内淋巴结转移、甲状腺癌肺内转移、阴茎癌肺内转移各1例。11例诊断为原发性肺癌的患者中，包括腺癌6例、鳞癌1例、小细胞癌3例、肉瘤样癌1例。14例排除恶性可能的患者中，包括反应性淋巴结炎9例、肺结核1例、类结节病反应4例。41例患者诊断流程详见图 1。

**1:**
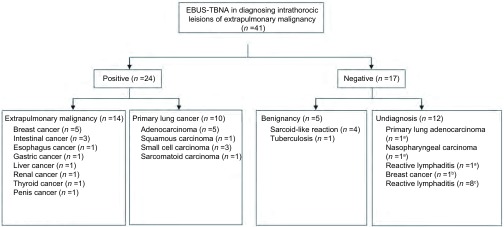
41例患者EBUS-TBNA诊断流程。EBUS-TBNA：气管内超声引导下的经支气管针吸活检；a：开胸手术证实；b：淋巴结活检证实；c：临床随访证实 Flowchart of 41 patients undergoing EBUS-TBNA. EBUS-TBNA: endobronchial ultrasound-guided transbronchial needle aspiration; a: confirmed by thoracotomy; b: confirmed by lymph node biopsy; c: confirmed by clinical follow-up

### 2.3 EBUS-TBNA诊断结果

16例最终诊断为肺外转移癌，其中14例通过EBUS-TBNA获得诊断，假阴性2例（乳腺癌胸内淋巴结转移、鼻咽癌胸内淋巴结转移各1例）。其中12例依赖免疫组化检测结果明确肿瘤细胞来源（代表病例见图 2）。11例最终诊断为原发性肺癌，10例经EBUS-TBNA诊断，包括腺癌5例、鳞癌1例、小细胞癌3例、肉瘤样癌1例，其中4例腺癌和1例肉瘤样癌通过免疫组化检测证实为原发性肺癌；假阴性1例。3例假阴性患者中，均TBNA 7组淋巴结，标本未见恶性依据，后经其他病理结果证实（图 1）。EBUS-TBNA对肺外肿瘤胸内转移和胸内良恶性病变诊断的敏感性及准确性分别为87.50%、88.89%和95.12%、92.68%（表 1）。

**2:**
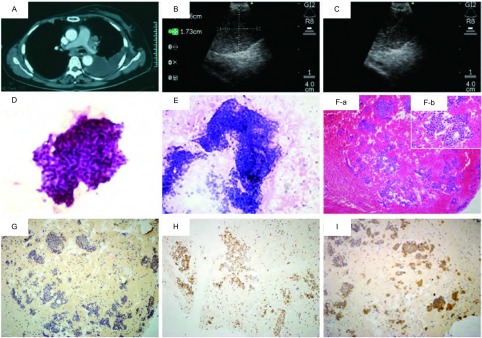
EBUS-TBNA诊断乳腺癌肺部转移。患者，女性，60岁，乳腺癌术后8年，因咳嗽气促1周伴恶心入院。A：胸部CT示右侧乳房术后改变，左侧胸腔积液，左肺门及隆突下LN增大；B：EBUS测量7 LN；C：EBUS-TBNA 7 LN；D：TBNA薄层细胞学检查（TCT）示腺癌（HE, ×40）；E：TBNA细胞学涂片示腺癌（HE, ×20）；F：TBNA活检组织病理学示腺癌（HE, a: ×10, b: ×40）；G：TBNA活检组织免疫组织化学染色TTF-1阴性（-）；H：TBNA活检组织免疫组织化学染色ER阳性（+）；I：TBNA活检组织免疫组织化学染色PR阳性（+）。LN:淋巴结 Breast cancer lung metastasis diagnosed by EBUS-TBNA in a 60-year-old female patient with breast cancer for 8 years. A: Chest computed tomography (CT) shows the right side of the breast postoperative change, left pleural effusion and the left pulmonary hilar and subcarinal lymph node enlarged; B: Measurement of 7 LN by EBUS; C: EBUS-TBNA 7 LN; D: TBNA thin layer cytology test (TCT) indicated adenocarcinoma (HE, ×40); E: TBNA cytological smear indicated adenocarcinoma (HE, ×20); F: TBNA tissue specimens pathology indicated adenocarcinoma (HE, a: ×10, b: ×40); G: TBNA biopsy immunohistochemical staining TTF-1 negative (-); H: TBNA biopsy immunohistochemical staining ER positive (+); I: TBNA biopsy immunohistochemical staining PR positive (+). LN: lymph node

**1:** EBUS-TBNA对肺外恶性肿瘤胸内病变的诊断价值 The diagnostic yield of EBUS-TBNA in the intrathoracic metastasis from extrapulmonary malignancy

	Sensitivity	Specificity	Positive predictive value	Negative predictive value	Accuracy
Metastatic carcinoma	87.50% (14/16)	100% (25/25)	100% (14/14)	92.59% (25/27)	95.12% (39/41)
Malignancy	88.89% (24/27)	100% (14/14)	100% (24/24)	82.35% (14/17)	92.68% (38/41)

9例临床表现为反应性淋巴结炎的患者中，EBUS-TBNA均排除恶性诊断，其中1例经术后病理证实为反应性淋巴结炎，8例经临床随访证实。4例患者为类结节病反应，ENUS-TBNA病理提示类上皮细胞形成的肉芽肿，均排除肺外转移癌和原发性肺癌，1例经纵隔镜手术明确，3例经密切随访证实。1例肺结核患者，EBUS-TBNA病理提示类上皮细胞肉芽肿伴坏死，TBNA标本微生物学检测找到结核分枝杆菌，后经规范抗结核治疗后病情好转。

EBUS-TBNA术前曾行PET/CT检查的20例患者中，19例SUV增高，1例SUV正常；EBUS-TBNA诊断肺外转移癌7例，原发性肺癌6例，炎症3例和类结节病反应2例，EBUS-TBNA未诊断（假阴性）2例（乳腺癌胸内淋巴结转移和原发性肺癌各1例）；最后诊断证实为肺外转移癌8例，原发性肺癌7例，炎症3例和类结节病反应2例。PET/CT和EBUS-TBNA对胸内病变良恶性诊断的敏感性、阳性预测值、准确率分别为93.33%（14/15）、73.68%（14/19）、70.00%（14/20）和86.67%（13/15）、100%（13/13）、90.00%（18/20）。

### 2.4 并发症

患者均能很好耐受EBUS-TBNA操作，除外1例高龄患者因肺功能差，第一次操作中仅穿刺一次，便出现低氧，停止操作后自行缓解，考虑与咪达唑仑应用相关，择日行第二次EBUS-TBNA检查，在单纯局部麻醉下顺利进行操作，得以诊断；操作过程中除内窥镜观察到穿刺点少许出血外，未发现气胸、纵隔气肿及纵隔大血管破裂出血等严重并发症。

## 3 讨论

肺外肿瘤发生胸内淋巴结转移或肺内转移通常提示晚期肿瘤，鉴别新发的胸内淋巴结增大或肺内病灶是胸外肿瘤的转移还是新发的第二原发肿瘤（例如肺癌），对患者的预后和治疗计划的制定具有重要作用。在另外一些患者由于放化疗等导致免疫力低下，新发的胸内淋巴结增大或肺内病灶可能是结核分枝杆菌或真菌感染，或是淋巴结对治疗或炎症及肿瘤的反应性炎症改变^[13]^。本项前瞻性研究入组41例患者，结果提示EBUS-TBNA在肺外恶性肿瘤胸内转移中的诊断准确率为95.12%，证实了EBUS-TBNA是评估肺外恶性肿瘤胸内病变安全、有效的检查手段。

目前EBUS-TBNA用于肺外肿瘤胸内转移诊断的研究相对较少，且均为回顾性研究^[14-20]^。Navani等^[16]^开展的一项多中心研究结果证实EBUS-TBNA在161例怀疑肺外恶性肿瘤胸内转移的患者中诊断敏感性为87%，而其他几项单中心研究的诊断敏感性为85%-96.3%^[14, 15, 17-20]^。本研究中EBUS-TBNA诊断肺外恶性肿瘤胸内转移的敏感度为87.50%，与文献报道结果类似。EBUS-TBNA诊断假阴性较高的问题，是目前临床应用EBUS-TBNA面临的难点之一，本研究中出现3例假阴性均出现在7组淋巴结，考虑该组淋巴结范围大，且包含多个淋巴结，从左、右侧支气管均可穿刺，穿刺针未进入发生癌细胞转移的淋巴结或转移淋巴结的转移区域可导致假阴性结果。因此笔者认为术前仔细评估CT或PET/CT，依据肿瘤转移特点，对肿大淋巴结进行重点全面穿刺以提高标本质量，可一定程度上减少假阴性率。且因肺外恶性肿瘤胸内转移的诊断较大程度上依赖免疫组化检测结果，获得足量和合格的标本进行后续的免疫组化检测尤为关键。

免疫组化检测在诊断肺外恶性肿瘤胸内转移具有重要意义，可以判定肿瘤起源，并能根据肿瘤的治疗标记物的免疫组化检测结果预测治疗对肿瘤反应^[14]^。例如，转移性乳腺癌患者雌激素受体（estrogen receptor, ER）和孕激素受体（progesterone receptor, PR）表达阳性的患者，内分泌治疗有效率高达80%，人类表皮生长因子受体（human epidermal growth factor receptor, HER-2）阳性者可选用靶向治疗药物曲妥珠单抗治疗。转移性腺癌可以通过选用不同的酶标来判断其来源，如转移性前列腺癌可见甲状腺转录因子（thyroid transcription factor-1, TTF-1）阴性、前列腺特异性抗原（prostate specific antigen, PSA）阳性、P504S阳性。转移性鳞癌目前并无好的免疫酶标可以判定，通常根据转移灶和原发灶的形态对比判断，通常转移性鳞癌与原发病灶形态学特征相似，但角化特征更明显，可作为判断转移的经验应用。

CT、PET/CT是判断肺外肿瘤发生胸内转移的常规手段，但对于短径小于1 cm的病变诊断的敏感性不够，对于短径大于1 cm的病变诊断的特异性不够，特别是胸内结核和淋巴结类结节病反应在PET/CT上也表现为FDG代谢增高，出现假阳性的结果。Song等^[15]^研究显示PET/CT对肺外恶性肿瘤胸内转移诊断的特异性和阳性预测值分别为83%和89%，而Ozgül等^[20]^在结核高发地区的研究显示PET/CT对肺外恶性肿瘤胸内转移诊断的特异性和阳性预测值分别为35.7%和66.6%。因中国为结核高发国家，需考虑PET/CT导致假阳性结果偏高的可能。本研究结果显示PET/CT对肺外肿瘤患者胸内病变良恶性诊断的特异性和阳性预测值低于EBUS-TBNA。表明EBUS-TBNA在肺外肿瘤患者胸内病变良恶性鉴别的特异性和阳性预测值方面有显著优势。

综上所述，EBUS-TBNA诊断特异性和准确率高于PET/CT，可以安全有效的为肺外恶性肿瘤合并的胸内病变提供病理学依据，结合免疫组化检测，可以判断恶性肿瘤来源，从而指导制定合理的诊疗方案。
